# Comment to: Propofol and survival: an updated meta-analysis of randomized clinical trials

**DOI:** 10.1186/s13054-023-04489-4

**Published:** 2023-05-24

**Authors:** David Benavides-Zora, Jose Hugo Arias-Botero

**Affiliations:** 1 Massachusetts General Hospital, Boston, USA; 2 Universidad CES, Medellín, Colombia

**Dear Editor,**

We read with delight the newly published meta-analysis on propofol and survival in different settings by Kotani et al. [1]. We want to congratulate the authors, which made a considerable effort to carry through a wealthy systematic review obtaining a significant number of studies to evaluate this critical question regarding propofol.

We will carefully dissect the three most significant limitations we encountered in the main result of the article that suggests a 10% increase in mortality of propofol when compared to other hypnotic agents in the overall population (RR = 1.10, 95% CI = 1.01–1.20, *p* = 0.03) [1].

## Pooling different time points in mortality

The authors choose as the primary outcome all-cause mortality at the longest follow-up available, pooling different time points for this outcome. They supported the use of varying timepoint in mortality by only the article by Roth et al., which found that in ICU clinical trials, you can pool risk ratios from different time points in mortality with a good chance that would not affect your results and could improve your N [2].

Although this is an exciting approach, these authors have only studied it in the context of ICU trials. The systematic review we are commenting on focuses more on the peri-operative setting, with some subgroup analyses in ICU trials.

Roth et al., in case of doubt, suggest, “*Furthermore, we advocate sensitivity analyses on mortality time point definitions if there is doubt on their influence in meta-analyses of particular interventions”* [2]*.* Kotani et al. [1] accurately performed a subgroup analysis (authors table S4) that showed statistical differences in the results of different time points. Considering these points, we highlight the reason not to pool 1-year mortality with shorter mortality time points.

Moreover, in these studies, the 1-year mortality outcome may be at high risk of bias due to dropout and non-protocol interventions. For example, in Likhvantsev's study, of the patients randomized (431 to propofol and 437 to volatile), the outcome could only be assessed in 292 and 326, respectively [3]. Therefore, we do not agree with assigning, for this outcome, a low risk of bias; this issue, beyond statistical considerations, raises epidemiological questions about attributing a direct effect of propofol on mortality.

## Pooling different settings

The authors performed a subgroup analysis for different settings. They presented results for cardiac surgeries, non-cardiac surgeries, and ICU. The results in Table 1 in the original article show an apparent statistical difference only in the cardiac surgery subgroup. Again, this would demonstrate that pooling these results altogether is not advisable due to high clinical heterogeneity. According to the Cochrane manual [4], clinical heterogeneity should be assessed, and beyond statistical heterogeneity, considering the pooling of studies in different settings, patient profiles, and types of interventions, we strongly suggest using a random-effects model instead of a fixed-effects model; which would represent more accurate the data provided and would show no statistical difference as described in authors figure S21.

Thoughtfully observing the Forest plot in Cardiac Surgery, the study by Likhvantsev et al. [3] is the only RCT not crossing the line of no effect, also carrying an overall 61% weight in the forest plot. Due to the importance of this article to the general pool analysis, we found an error in the data entry. Likhvantsev et al. found mortality in the sevoflurane and propofol group, respectively, of 52/292 and 81/326, compared to 52/450 and 81/450, showed in Kotani’s metanalysis. This could lead to an overestimation of the effect and weight of this study on the pooled estimator.

## Spin

“Spin” refers to presenting a result as beneficial or detrimental even though the results do not support it. It has become a common practice, achieving roughly 40% of every RCT in the anesthesia literature [5]. To ensure accurate research interpretation and dissemination, “spin” language in the discussion when there is no statistical difference, such as “direction and magnitude” or “trend toward an increased mortality,” should be avoided. If the authors did not find any statistical differences in the ICU setting, we consider it premature to launch a recommendation as, “*While waiting for large, randomized trials, we suggest physicians consider alternative hypnotic agents when available and feasible, to implement hypnotic rotation strategies in the ICU, and to attempt propofol dose reduction whenever is possible.”*

We agree with the need for studies evaluating the effect of propofol on relevant clinical outcomes in more homogeneous settings. Due to the substantial implications that this article suggests and with the stakes as high regarding this matter, we believe that the readers of *Critical Care* would benefit from the suggestions and edits mentioned.

## Data Availability

The original data were obtained from the manuscript and supplemental material provided by the Journal.
